# Bibliographical Notices

**Published:** 1839-06-29

**Authors:** 


### BIBLIOGRAPHICAL NOTICES.

*Address to the Graduates of the Philadelphia College of Pharmacy, April* 23*d*, 1839. By Joseph Carson, M. D., Professor of Materia Medica and Pharmacy.

The character of the pharmaceutic profession in Philadelphia is deservedly high. Since its disconnection with the sister profession of physic—a connection still generally maintained throughout the country—it has fallen into excellent hands. Our apothecaries have the benefit of a course of capital lectures, and commence their avocation with a certificate of attainments, upon which, from the character of the examination to which they are subjected, the profession and the community may rely with confidence. The address before us, delivered at the last annual graduation, is a well conceived and executed portraiture of pharmacy, as it should be, as a profession and a science.

---

*The Invalid's Guide to the Hot Springs of Virginia, with Cases, &c.* By Thomas Goode, M. D.

The Hot Springs of Virginia consists of six springs, the temperature of which ranges from 98 to 106 degrees of heat. Taken internally, they are antacid, aperient, diuretic, and diaphoretic. They are chiefly efficacious, however, when used as a bath. Dr. Goode publishes a number of cases, illustrative of their good effects in a variety of diseases.

---

*Proceedings of the Medical Convention of Ohio, held May*, 1839. Cleveland, 1839.

The scientific and professional zeal of our brethren of the West again elicits our applause, in the proceedings of the third session of the Medical Convention of the State of Ohio. The perusal of the Journal of these proceedings has given us great pleasure, and strengthened our conviction of the numerous happy effects which result from such assemblages. Why cannot the Profession of our own state follow the example of our enterprising sisters ?—we trust that she will, at least, respond to the call for a National Medical Convention, to which the Convention of Ohio has elected delegates, to meet in Philadelphia, in May, 1840. Two interesting papers were read at the Ohio Convention—an elaborate and spirited sketch of the climate and early diseases of Ohio, by Dr. Hildreth, and a more particular account of the medical topography of Licking county, by Dr. Richards. Productions like these illustrate the value of Medical Conventions.

